# Quality of life in patients with schizophrenia

**DOI:** 10.1192/j.eurpsy.2021.1454

**Published:** 2021-08-13

**Authors:** S. Hentati, M. Ben Abdallah, M. Daoud, M. Bouhamed, I. Baati, R. Sallemi, I. Feki, J. Masmoudi

**Affiliations:** 1 Psychiatry A, hedi chaker hospital, Sfax, Tunisia; 2 Department Of Psychiatry (a), CHU Hedi CHaker hospital Sfax Tunisia, Sfax, Tunisia; 3 Psychiatry, Hedi chaker hospital, Sfax, Tunisia; 4 Psychiatry A, CHU Hédi Chaker Sfax, Sfax, Tunisia

**Keywords:** schizophrénia, quality of life

## Abstract

**Introduction:**

Schizophrenia, with its high prevalence, chronic progression and social impact, is a major challenge for health professionals. For this reason it is important to assess the impact of this disease on these patients, mainly on their quality of life.

**Objectives:**

To study the quality of life in patients with schizophrenia and determine the factors correlated with it.

**Methods:**

A cross-sectional, descriptive and analytical study of 28 patients with schizophrenia followed up at the psychiatric consultation in Hédi Chaker University Hospital of Sfax. Data collection was performed using a sheet exploring socio-demographic and clinical data. We used the Quality of Life Scale (Q-LES-Q-SF).

**Results:**

The average age of our patients was 40.61±6.27 years. The sex ratio (M/F) was1.15. The socioeconomic level was low in 71.4%. The average number of relapses was 3.04±1.4. Follow-up and compliance were good in 28.6% of cases. The average number of hospitalizations was 3.04±1.4. The average of quality of life in patients with schizophrenia was 21±5.74. The quality of life was affected with age (p=0.023), with the high number of relapses and with a higher number of hospitalizations in psychiatric hospital (p=0.008). Quality of life was improved with regular follow-up and good adherence to the treatment (p=0.000).

**Conclusions:**

The quality of life in mental disorders was impaired mainly in schizophrenics, hence the need to evaluate in a codified way the quality of life of our patients in order to raise awareness among general practitioners as well as psychiatrists to improve the therapeutic and social care of patients.

